# Aptamer Therapeutics in Cancer: Current and Future

**DOI:** 10.3390/cancers10030080

**Published:** 2018-03-19

**Authors:** Yoshihiro Morita, Macall Leslie, Hiroyasu Kameyama, David E. Volk, Takemi Tanaka

**Affiliations:** 1 Stephenson Cancer Center, University of Oklahoma Health Sciences Center, 975 NE 10th, BRC-W, Rm 1415, Oklahoma City, OK 73104, USA; yoshihiro-morita@ouhsc.edu (Y.M.); Macall-Leslie@ouhsc.edu (M.L.); hirokameyama@dent.osaka-u.ac.jp (H.K.); 2 McGovern Medical School, Institute of Molecular Medicine, University of Texas Health Science Center at Houston, 1825 Hermann Pressler, Houston, TX 77030, USA; David.Volk@uth.tmc.edu; 3 Department of Pathology, College of Medicine, University of Oklahoma Health Sciences Center, 940 SL Young Blvd, Oklahoma City, OK 73104, USA

**Keywords:** aptamer, cancer, targeted therapy

## Abstract

Aptamer-related technologies represent a revolutionary advancement in the capacity to rapidly develop new classes of targeting ligands. Structurally distinct RNA and DNA oligonucleotides, aptamers mimic small, protein-binding molecules and exhibit high binding affinity and selectivity. Although their molecular weight is relatively small—approximately one-tenth that of monoclonal antibodies—their complex tertiary folded structures create sufficient recognition surface area for tight interaction with target molecules. Additionally, unlike antibodies, aptamers can be readily chemically synthesized and modified. In addition, aptamers’ long storage period and low immunogenicity are favorable properties for clinical utility. Due to their flexibility of chemical modification, aptamers are conjugated to other chemical entities including chemotherapeutic agents, siRNA, nanoparticles, and solid phase surfaces for therapeutic and diagnostic applications. However, as relatively small sized oligonucleotides, aptamers present several challenges for successful clinical translation. Their short plasma half-lives due to nuclease degradation and rapid renal excretion necessitate further structural modification of aptamers for clinical application. Since the US Food and Drug Administration (FDA) approval of the first aptamer drug, Macugen^®^ (pegaptanib), which treats wet-age-related macular degeneration, several aptamer therapeutics for oncology have followed and shown promise in pre-clinical models as well as clinical trials. This review discusses the advantages and challenges of aptamers and introduces therapeutic aptamers under investigation and in clinical trials for cancer treatments.

## 1. Advantages of Aptamers

Molecularly targeted therapy is broadly adopted for treatment of many cancer types as an opportunity to inhibit oncogene function. Currently, chimeric monoclonal antibodies as well as small molecule inhibitors are the clinical mainstays in this class of agents. Aptamers, or so called “chemical antibodies,” represent a new class of molecular targeting agents as a result of their unique properties, such as ease of synthesis and modification as well as high affinity binding and excellent safety profiles.

The structural base of aptamers is composed of short DNA or RNA oligonucleotides ranging around 15–100 nt that form complex tertiary or quadruplex structures through hybridization of complementary sequences [1]. Large surface areas, despite their small molecular weight (5–30 kDa), permit high-affinity binding to their molecular targets [1]. The dissociation constant (Kd) of an aptamer’s target is generally in the range of several micro- to pico-molars [2,3], which is comparable to antibody therapeutics. In contrast, small molecule inhibitors (Tyrosine kinase inhibitors: TKI) function as ATP mimetics, thus their sizes are small enough to occupy the ATP binding pocket of the intracellular domain of a receptor tyrosine kinase and are responsible for their relatively large Kd [4]. For example, gefitinib (Iressa^®^, AstraZeneca, Chesire, UK), the TKI for EGFR, binds with wild-type EGFR kinase at a Kd of 53.5 nM [5], while the Kd of cetuximab (Erbitux^®^, ImClone, Branchburg, NJ, USA), an inhibitory anti-EGFR antibody, is far smaller, 2.3 nM [6]. A nuclease resistant 2′-fluoropyrimidines-containing RNA aptamer, named CL4 and E07, a 2′-fluoropyrimidine modified anti-EGFR aptamer display Kd comparable to antibody, at 10 nM [7] and 2.4 nM, respectively [8].

Aptamer backbones are synthesized automatically through cell-free assembly that enables cost effective and rapid bulk production with minimal batch-to-batch variation. Additionally, aptamers’ structural stability affords them an extensive storage period as well as the ability to withstand a broad range of temperatures. They are stable at ambient temperature and heat resistant, thus their functional tertiary structure is readily regenerated following heat denaturation. Another significant advantage of aptamers is their capacity for site-specific chemical modifications. Oligonucleotide sugar, base, and phosphate backbone modifications as well as a variety of unnatural oligonucleotides make up the wide repertoire of chemical alterations available to aptamers. Methods for base substitutions including 2′-fluoro- [9,10,11], 2′-amino-, 2′-azido-, 2′-hydroxymethyl-, and 2′-methoxypyrimidines and 2′-methoxypurines have been established [12,13,14,15]. Phosphorothioate and phosphorodithioate substitutions are another option for the backbone modification [16]. Such chemical modifications of the DNA backbone provide resistance against nucleases, as was first shown by Eckstein’s group [17], and often increase binding affinity [16]. The introduction of functional groups in the aptamer backbone permits conjugation to other drugs, siRNA [18,19], and nanoparticles [20,21,22], further broadening their application as multivalent therapeutics [23,24,25,26,27,28,29,30].

## 2. Challenges and Possible Solutions in Aptamer Therapeutics

### 2.1. Aptamer Stability

For treatment of malignancies, it is ideal that drugs remain in circulation for extended periods to increase chance of cancer cell exposure to drugs. Thus, the pharmacokinetic profile and bioavailability of injectable drugs are critical determinants of therapeutic efficacy. In this respect, humanized antibodies are superior to other drug entities, displaying circulation half-lives from days to weeks [31]. Unmodified nucleotides, however, may have a serum half-life as short as few minutes [32]. This unfavorable pharmaceutical property represents one the critical challenges facing realistic clinical application of aptamers. Two contributing factors for this are their susceptibility to nuclease degradation and renal excretion. Nucleases are abundantly present in biological fluids, and both *exo*- and *endo*-nucleases cleave phosphodiester bonds of single and double stranded oligonucleotides [1]. The average time of oligonucleotide decay in the blood depends on their structure, and ranges from several minutes to several tens of minutes [1]. Since such a short half-life is undesirable for therapeutic applications, oligonucleotides are typically chemically modified to improve the serum stability [3]. For instance, simple alteration of the 2′-OH group on ribose to 2′-amino (NH_2_) or 2′-fluoro-modified sugars on pyrimidines can successfully confer resistance to breakdown in the serum for over 2 days [33] and remains present in the eyes after 28 days [34]. Thus, current clinically used aptamers [35] employ chemical modifications such as replacing 2’-OH with fluoro [8,36], NH_2_ [13,37], or O-methyl [38,39] groups at the 2′ position or capping the 3′ end with inverted thymidine to overcome nuclease degradation. Anti-human TNFα RNA aptamer which exhibits increased stability with the 2′-NH_2_ and 2′-fluoro modifications is one example using these methods. While degradation half-life of the unmodified TNFα RNA aptamer in the serum was several minutes, 2′-NH_2_ and 2′-fluoro modifications extended to 8 h [13]. Another study indicated that a RNA aptamer with 2-O-methyl-modification of all nucleotides in the backbone was stable in RNase rich mycoplasma-contaminated media for more than 4 h, whereas the stability of the modification on pyrimidines only was less than 30 min [39]. Thiophosphate-substituted aptamers, called thioaptamers, enhance stability by replacing one (monothioaptamer) or both (dithioaptamer) of the non-bridging phosphoryl oxygens in the oligonucleotide phosphate backbone with sulfur [40]. In addition to stabilization, thioation enhanced binding affinity as well, owing to decreased negative charge on DNA backbone [40]. Dithioaptamer developed by Gorenstein’s group [16] showed an increased binding affinity up to 28–600 fold compared to monothioaptamer [41]. “Mirror aptamers” (Spiegelmers) with oligonucleotide backbones composed of l-ribose (RNA Spiegelmers) or l-deoxy-ribose (DNA Spiegelmers) also lead to nuclease resistance. Spiegelmers, l-form aptamers that are natural d-form chiral inversions are not recognized by nucleases, and therefore display increased stability compared to their natural d-nucleic acid counterparts [42]. Pfander et al. developed a novel initiator nucleotide that resulted in an aldehyde modification of the 5′-end of RNA, which can be further modified with amino- or hydrazine-functionalized groups [43]. A38, an unmodified DNA aptamer for Vaccinia virus, also exhibits high stability [44] that may result from three-dimensional structures protecting the 3′- and 5′-termini of the aptamer from exonucleases. Examples of unmodified aptamer structures that confer high nuclease degradation resistance include closed ring structures at both terminal ligations or ligation of several different aptamers, forming closed structures with multiple specificities [45,46]. The degradation half-life of unligated “open” TS-1 aptamer (DNA aptamer bearing L-selectin-hairpin head) was 4.8 and 16 h in pooled human serum and plasma respectively, while the ligated “closed” TS-1 aptamer significantly increased the stability against nucleases to 9.5 and 50 h, respectively [45].

### 2.2. Renal Excretion

The primary excretion routes of injectable drugs are through the kidneys and liver. With aptamer sizes ranging from 5 to 30 kDa, the average diameter of aptamers is less than 5 nm [47], so most aptamers are smaller than the renal filtration threshold of glomeruli (i.e., 50 kDa) and are excreted from the kidney, regardless of their nuclease resistance. One common solution to overcome rapid renal excretion is augmentation of the aptamer’s overall size through conjugation with high molecular weight moieties such as polyethylene glycol (PEG) [48], cholesterol [49,50], protein [51,52], multimerization [53,54,55], or nanomaterials [56,57,58]. PEG is a US Food and Drug Administration (FDA) approved [59] hydrophilic biocompatible material that has been adopted in 12 biopharmaceuticals currently marketed for human use [60]. PEG is commercially available in a variety of sizes (0.3 to 10,000 kDa), with different terminal functional groups for chemical conjugation. For aptamer application, PEG is widely used for enlargement of the size [61] as well as addition of stealth effect to increase their retention in circulation. Macugen^®^ (OSI Pharmaceuticals, Melville, USA) is composed of a RNA aptamer backbone conjugated with 40 kDa PEG, and exhibits prolonged plasma half-life of 9.3 h via intravenous injection, 12 h via subcutaneous injection [33], and 94 h via vitreous humor [34]. Similarly, conjugation of 40 kDa PEG to a 2′-fluoro-/2′-*O*-methyl modified aptamer resulted in an increase of the circulation half-life to 23 h in a mouse model [48]. However, high molecular weight PEG conjugation may cause a functional hindrance of the aptamer binding to its target [62]. Thus the size of PEG as well as type and length of linkage of PEG to aptamer should be determined in a case-by-case fashion. Cholesterol and sugar-based polymers are alternative options for this purpose. Cholesterol conjugation to factor IXa specific aptamer prolonged its half-life significantly in pigs, increasing from only 5 to 10 min for the unmodified aptamer up to 1 to 1.5 h for the conjugated ones [49].

### 2.3. Safety of Aptamers

Several factors including aptamer sequence, dose (single and cumulative), route, and chemical modifications may contribute to adverse effects [63]. Intravenously administered aptamers are generally well tolerated; however, a few adverse effects related to dose and sequence have been reported [64,65]. Studies have shown that intravenously administered aptamers diffuse rapidly into the tissues of various organs, accumulating primarily in the liver, kidney, and spleen [66]. Accumulation of oligonucleotides in various tissues has been qualitatively measured by immunohistochemistry, in situ hybridization, or histopathology [67,68]. A study by Geary et al. found the accumulation of human TNFα antisense phosphorothioate aptamer in the kidneys, livers, lymph nodes, and spleens of mice and primates [66]. Administration of aptamers via peritoneum (100 mg/kg) and vein (50 mg/kg) caused reversible kidney and liver abnormalities in mice with a recovery period of 4 to 13 weeks [69,70,71]. Infiltration of mononuclear cells and hypertrophy of Kupffer cells in the liver as well as basophilic granules in the cytoplasm of tubular epithelial cells in the kidneys were reported [71,72,73].

Liver metabolism of oligonucleotides may result in hepatotoxicity. Kang et al. reported no definitive evidence of sinusoidal dilation, Kupffer cell hypertrophy, or perivascular inflammatory infiltrate in the liver after intravenous bolus injection of E-selectin antagonistic thioaptamer (ESTA) at a dose up to 500 μg twice weekly for 4 weeks, compared to saline injected control mice [74]. A slight elevation of transaminases aspartate aminotransferase (AST) and alanine aminotransferase (ALT) was noted, although the levels were still within normal range. Similarly, Phase I and II clinical trials of mipomersen, a phosphorothioate antisense oligonucleotide for apolipoprotein B synthesis inhibitor, showed transient dose-dependent elevation of AST and ALT over 5-week treatment (30–400 mg/week) [75,76]. In a randomized, placebo-controlled, dose-escalation Phase II study of mipomersen for 5–13-week treatment, an increase of ALT was noted among 17% of patients (10 of 59 cases), to a level more than three times the upper limit of the normal range [76]. In phase III trials, elevation of ALT with grade 2–4 (3-fold greater than upper limit of normal) was observed in 17.9% of patients (28 out of 156) who received mipomersen for 26 weeks treatment compared to the placebo arm [77,78,79,80].

Unintended anticoagulation effects have also been reported in ISIS 2302, a 20-mer antisense phosphorothioate oligonucleotide targeting human intercellular adhesion molecule-1 (ICAM-1) mRNA [81]. Prolongation of prothrombin and thrombin coagulation times was observed in in vitro assays in human plasma at concentrations of ISIS 2302 >100 μg/mL. Additionally, in a placebo-controlled trial of ISIS 2302 for the treatment of Crohn’s disease, intravenous infusion of ISIS 2302 (2 mg/kg) over 2 h caused a transient (2–4 h after dosing) increase in the activated partial thromboplastin time by approximately 10 seconds, compared to saline placebo infusion [82]. However, no increase in bleeding time was noted. 

Complement activation through the interaction of oligonucleotides has been reported. Repeated bolus intravenous injections of PEGylated E-selectin thioaptamer at a dose of 128 μg resulted in a 2-fold increase of plasma C3a level in mice compared with saline injected mice, though the level of increase was far less than the level of symptomatic hypersensitive reaction [62]. Henry et al. reported that a single dose of intravenous 10-min infusion of 20 mg/kg ISIS 2302 caused an increase of complement split products Bb and C5a (100- and 7-fold, respectively), accompanied by changes in blood pressure, neutrophil count, and serum cytokine levels (i.e., increases in IL-6, MCP-1, and IL-12) in primates. These changes were observed at or near the end of infusion, which led to death in one of the three primates approximately 4 h after infusion. This study demonstrated that complement pathway activation in primates was related to rapid infusion of phosphorothioate oligonucleotides [83]. Co-administration of CAB-2m, a complement activation inhibitor, alleviated complement pathway activation along with other clinical symptoms [84].

Since aptamers are synthetic nucleic acids, it is possible for them to be recognized by the innate immune system via germline-encoded-pattern-recognition receptors (PRRs) such as Toll-like receptors (TLRs) and retinoic acid-inducible gene (RIG)-I-like receptors (RLRs) [85]. Activation of Toll-like Receptor 3 (TLR 3), TLR 7, TLR 8 or TLR 9 induces immunity upon recognizing pathogen-derived nucleic acids [86]. Accordingly, TLR9 responds to unmethylated CpG motifs in DNA, TLR3 to double-stranded RNA, and TLR7 and TLR8 to single-stranded RNA [87]. Studies showed that the endocytosis of unmethylated CpG motif of DNA into dendritic cells or B cells expressing TLR9 triggers both innate and adaptive immune responses [88,89] and induces inflamatory cascades [88,89,90]. The CpG motif consists of an unmethylated CpG dinucleotide flanked by particular base contexts, such as two 5′-purines and two 3′-pyrimidines [91]. For example, the CpG motif with the “TCAACGTT” sequence was shown to induce B cell activation, whereas a similar sequence, “TCATCGAT”, had no effect on B cell activation. Interestingly, immunogenicity was either absent or limited even if pegaptanib doses 1000-fold higher than clinically required were intravenously administered to primates [92,93]. While development of antibodies against synthetic oligonucleotides is uncommon [93], it is noteworthy that antibodies to PEG may cause an adverse effect due to frequent exposure to PEGylated products [94]. In a phase II trial of pegloticase with 10 kDa PEG for refractory gout at a dose of 8 mg intravenous infusion every 3 weeks, 13 of 30 (43%) patients with pre-existing antibody against PEG experienced more frequent infusion reactions (eight of 13 patients, 62%) than patients without (five of 17 patients, 29%) [95].

## 3. Aptamers as Therapeutics under Investigation in Oncology

**Figure 1:**
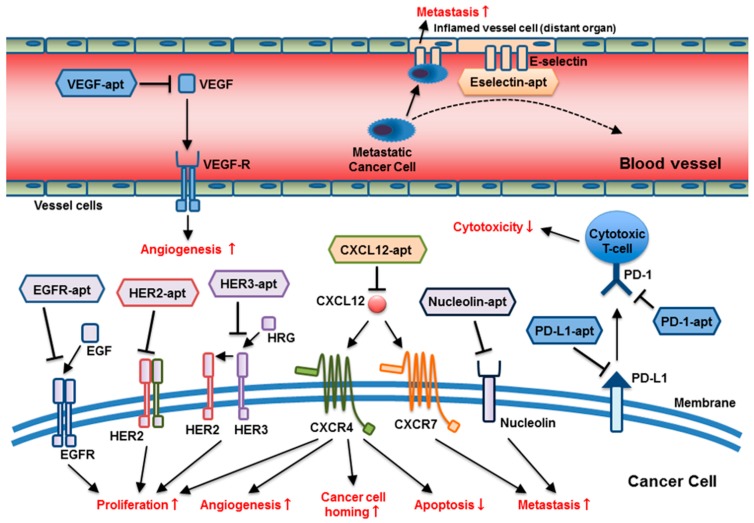
The broad range of molecular targets and targeting mechanisms of anti-cancer aptamers: Aptamers target multiple molecular pathways involving tumor progression and metastasis, including cancer cell proliferation, cell homing, apoptosis suppression, metastasis, impairment of T-cell cytotoxicity, and angiogenesis at different locations. (apt = aptamer).

The main strategy of aptamer-based therapeutics in oncology application is a blockade of protein-protein or receptor-ligand interactions as an antagonist. Accordingly, aptamers have been utilized as antagonists against oncoproteins or their ligands, targeting moieties for drug delivery in multivalent therapeutics. In this section, we specifically focus on therapeutic aptamers with antagonistic functions under pre-clinical investigation for anti-cancer therapy (Figure 1 and Table 1). In addition, antidote aptamers for controlled therapy are also discussed.

### 3.1. Aptamers as Tools for Cancer Therapy

#### 3.1.1. RNA Aptamer Targeting VEGF

Although the VEGF targeted aptamer pegaptanib is currently approved for use in ophthalmology (see the Clinical Trials section), it was originally designed for use in cancer treatment [148,149]. The cancer treatment strategy of anti-VEGF aptamer is inhibition of VEGF-associated tumor vessel formation by binding to VEGF thus blocking the VEGF/VEGF-R interaction [29]. Daily treatment of mice bearing human A673 rhabdomyosarcoma cell xenograft tumor with anti-VEGF PEGylated RNA aptamer (EYE001) at a dose of 10 mg/kg inhibited tumor growth by 74% relative to the control [149]. Recently, an aptamer-antibody hybrid complex has been prepared to improve the pharmacokinetics, using cotinine-specific antibody and cotinine-binding pegaptanib aptamer [52]. This hybrid complex showed prolongation of half-life in serum to 8.2 h. In an A549 (human lung adenocarcinoma cells) xenograft mouse model, systemic administration of the pegaptanib-antibody complex inhibited tumor angiogenesis and reduced tumor growth (<50%) compared to the human-IgG control mice group. This effect was comparable to that observed for bevacizumab, an anti-VEGF monoclonal antibody. 

#### 3.1.2. Aptamers Targeting the Epidermal Growth Factor Receptor (EGFR)

EGFR (ErbB1) is a receptor tyrosine kinase (RTK) that is often mutated and overexpressed in many types of solid tumors, resulting in tumor growth [150]. Thus, EGFR serves as an excellent therapeutic target, and anti-EGFR antibodies as well as TKI have been widely adopted for clinical use to block the downstream signaling cascade [150]. Similar to anti-EGFR antibody, the binding of anti-EGFR aptamer to the extracellular domain of EGFR blocks subsequent phosphorylation and downstream signaling, i.e., PI3K/AKT and MAPK signaling [29]. Several anti-EGFR aptamers have been developed and demonstrated antitumor effect in various cancers (vulvar carcinoma, lung carcinoma, breast cancer, glioblastoma, and epidermoid carcinoma) [7,8,108,109,110]. Esposito et al. reported that CL4, an anti-EGFR 2′-fluoro pyrimidine RNA aptamer, inhibited EGFR-mediated signal pathways, induced apoptosis, and inhibited tumor growth in a mouse xenograft model of human non-small-cell lung cancer (NSCLC) [7]. Li et al. identified an anti-EGFR 2′-fluoro pyrimidine modified aptamer (E07) that binds both wild-type EGFR as well as EGFR variant III (EGFRvIII), the most common deletion mutant of extracellular domain mutations that precludes ligand binding. The binding of E07 to EGFR expressing A431 epidermoid carcinoma cells led to inhibition of EGFR autophosphorylation and cell proliferation in three-dimensional cultures [8]. 

#### 3.1.3. Aptamers Targeting HER2 and HER3

HERs including HER2 (ErbB-2), HER3 (ErbB-3), and HER4 (ErbB-4) are a part of the EGFR family and transduce growth-promoting signals in response to dimerization of extracellular domains upon ligand binding [151]. Overexpression of HER2 is observed in approximately 10–15% of breast cancers and currently monoclonal antibody or TKI is used to block its function. Weekly intraperitoneal injection of trimeric DNA anti-HER2 aptamer at a dose of 40 μg exhibited a 2-fold higher tumor inhibitory effect on N87 human gastric cancer cells implanted in CD-1 mice compared to the HER2 monoclonal antibody (Ab431, 160 μg) [112]. In a recent study, HER3 has been shown to play a role in mediating resistance to HER2 and phosphoinositide 3-kinase (PI3K) pathway-directed therapies due to its feedback regulation via AKT signaling [152]. As a result, HER3 is being examined as a direct therapeutic target [152]. The first RNA aptamer against HER3 (A30) has been shown to block interaction between HER3 and its ligand (HRG; heregulin). Binding of HRG disrupts HER3 oligomerization and leads to the formation of signaling-competent heterodimers, preferentially with HER2. Binding of A30 to HER3 inhibited HRG-dependent tyrosine phosphorylation of HER2 and the HRG-induced growth response of MCF7 breast cancer cells in vitro [153]. 

#### 3.1.4. DNA Aptamer Targeting Nucleolin 

The abundant non-ribosomal protein, nucleolin, which shows abnormally increased cell membrane localization in several types of cancer, interacts with key oncogenes and plays an integral role in cellular proliferation, invasion, and apoptosis [100,154,155]. Nucleolin expressed on the cell surface interacts with ligands implicated in cell differentiation, survival, inflammation, angiogenesis, and tumor development [156]. AS1411 is a guanine quadruplex aptamer that binds to nucleolin, inducing growth inhibition in vitro and in vivo human xenograft models (renal cancer, lung cancer, MX1 breast cancer, and pancreatic cancer) [100]. Notably, AS1411 was discovered by screening for anti-proliferative activity among antisense oligonucleotides rather than the SELEX method typical in aptamer development [100]. The underlying mechanism of nucleolin-associated anti-proliferative effect involves binding to cell surface nucleolin, internalization of the aptamer-nucleolin complex, subsequent cell cycle arrest, and apoptosis [100,155]. AS1411 is currently in clinical trials for treatment of leukemia and renal cell carcinoma (see the Clinical Trials section) and is in pre-clinical investigation for other hematologic and solid malignancies. Recent in vitro studies have found that AS1411 displays activity against lymphoma through blockade of secondary targets (nuclear factor-κB and B-cell lymphoma 2) [157,158] and that in combination therapy with doxorubicin it reduced diffuse large B-cell lymphoma (DLBCL) cell survival [159]. Xenograft tumor models derived from A498 renal cancer, SKMES lung cancer, and MX1 breast cancer cells showed that intravenous administration of AS1411 delays tumor growth [100]. Moreover, combination therapy with AS1411 and gemcitabine, a chemotherapeutic agent used to treat pancreatic and non–small cell lung cancer in clinical practice, resulted in an increased antitumor effect compared to gemcitabine alone in xenograft model of human pancreatic cancer [100].

#### 3.1.5. DNA Aptamer Targeting PD-1/PD-L1

Programmed cell death 1 (PD-1; also known as CD279) is expressed in several cell types including T-lymphocytes, specifically CD8 tumor-infiltrating lymphocytes, which are in charge of directly eradicating tumor cells [160]. The interaction between PD-1 expressed on the surface of T-lymphocytes and PD-L1 expressed on cancer cells leads to an impairment of CD8 cytotoxicity. Prodeus et al. developed a 40 kDa PEGylated DNA aptamer, PEG-MP7, that specifically binds to PD-1, decreases tumor growth, and increases survival in mouse tumor models [139]. Their study found that PD-L1-mediated suppression of IL-2 secretion was functionally inhibited in primary T-cells treated with the base aptamer MP7 or a known anti-PD-1 antibody, but not control aptamer. Furthermore, an in vivo study using a murine colon cancer model (MC38 cells stably expressing human CEA; MC38.CEA) showed that PEG-MP7 effectively inhibited PD-1: PD-L1 interaction, showing efficacy equal to anti-PD-1 antibody in suppressing PD-L1+ carcinoma cell growth. Additional study by Lai et al. reported the PD-L1 antagonizing DNA aptamer (aptPD-L1) for the blockade of the binding between human PD-1 and PD-L1 [140]. Repeated intraperitoneal administrations of aptPD-L1 (1.2 mg/kg, 4 times a week) led to significant tumor growth inhibition compared to random-sequence aptamer administration in both the CT26 colon cancer cell line and LL/2 Lewis lung cancer cell line murine syngeneic tumor model mice.

#### 3.1.6. RNA Aptamer Targeting SDF-1 (CXCL12)

CXCL12 (C-X-C motif chemokine ligand 12), also known as SDF-1 (stromal cell-derived factor-1), is a critical chemokine involved in tumor metastasis, angiogenesis, cancer cell homing, and proliferation [161,162]. NOX-A12 is a PEGylated mirror-image RNA aptamer that displays high binding affinity to CXCL12 at a Kd of 0.2 nM [104]. Through binding to CXCL12, NOX-A12 inhibits signaling on both its receptors, CXCR4 and CXCR7, thus preventing angiogenesis as well as tumor cell proliferation, invasion, and metastasis [104]. Based on the efficacy of NOX-A12 as a treatment in a pre-clinical study of hematologic malignancies, it is currently in clinical trials for treatment of leukemia (CLL) and multiple myeloma (see Clinical Trials section). In addition, CXCL12 is expressed in the tumor microenvironment mainly by cancer-associated fibroblasts [163] and has been indicated for resistance to checkpoint inhibitors such as anti-PD-L1 through T-cell exclusion [164]. Zboralski et al. showed that NOX-A12 broke the immune-privileged status of the tumor microenvironment by paving the way for immune effector cells to enter into the tumor [107]. In this study, combination of NOX-A12 and anti-PD-1 (NOX-A12: 20 mg/kg s.c., every other day, anti-PD-1: 10 mg/kg i.p. twice weekly) resulted in significant tumor size reduction, superior to either vehicle, anti-PD-1 monotherapy, or NOX-A12 monotherapy in a murine syngeneic CT-26 colon cancer model.

#### 3.1.7. Thioaptamer Targeting E-Selectin

E-selectin is an adhesion molecule expressed on the luminal surface of inflamed blood vessels which mediates hematogenous metastasis by assisting shear-resistant adhesion of circulating tumor cells to the inflamed vessel surface under dynamic blood flow [128]. An E-selectin antagonistic thioaptamer (ESTA) identified from cell-SELEX demonstrated the ability to inhibit adhesion of circulating cells to E-selectin expressing endothelial cells with binding affinity and IC50 of 47 nM and 63–83 nM, respectively. This accounts for >10,000 times higher affinity and 1000 times lower IC_50_ to its natural ligands (sLe^x^, Kd = 100–2000 uM; IC50 = 100–750 uM) [128]. Accordingly, a single intravenous injection of ESTA effectively prevented hematogenous metastasis of CD44^high^ breast cancer cells to a level equal to baseline by abrogating their adhesion to E-selectin-expressing pre-metastatic vascular niches in both syngeneic and xenogeneic forced breast cancer metastasis models [129]. Additionally, truncated ESTA conjugated with 10 kDa PEG extended its serum half-life and led to improvement of its anti-metastasis activity compared to parental ESTA [62]. Additionally, twice weekly intravenous administration of PEGylated ESTA at a 128 μg dose was well tolerated; no symptomatic changes in the ALT or AST levels, C3a, C5a complement levels, inflammation, or tissue damage (kidney, lung, or heart) were noted. Based on its E-selectin specific binding ability, ESTA was also used as a targeting moiety for nano- and microparticles [165]. ESTA conjugation to liposome enhanced tumor targeting in mouse models of breast cancer and conjugation of ESTA to porous silicon microparticles allowed bone marrow targeting in mice [166,167]. This exemplifies the versatile applications of aptamers as therapeutic agents and targeting moieties.

#### 3.1.8. RNA Aptamer Targeting CD40

CD40Apt, a 2-fluoro-RNA aptamer against CD40, has shown antitumor effect on CD40-expressing A20 lymphoma cells in vitro and in vivo. Soldevilla et al. reported that CD40Apt-SMG1-shRNA chimera improved survival of BALB/c mice that were intravenously inoculated with A20 lymphoma cells compared to CD40Apt-control-shRNA or untreated mice [145].

#### 3.1.9. DNA Aptamer Targeting CTLA-4

Monoclonal antibodies targeting cytotoxic T lymphocyte antigen-4 (CTLA-4) and PD-1/PD-L1 axis are now a part of routine clinical practice [168]. Huang et al. reported a novel high-affinity CTLA-4-antagonizing DNA aptamer that promoted lymphocyte proliferation and inhibited tumor growth in a murine syngeneic tumor model with mouse TC-1 lung cancer cells [146].

#### 3.1.10. RNA/DNA Aptamer Targeting C5a

A synergistic antitumor effect through inhibition of C5a/C5a receptor-1 and PD-1 signaling has been reported. Ajona et al. showed that blockade of PD-1 via RMP1-14 antibody and signaling inhibition of complement C5a/CD5a receptor via AON-D21 L-aptamer, reduced tumor growth and metastasis in syngeneic models of lung cancer. This study further showed a complete reversal of CD8 T-cell exhaustion contributed prolonged survival in mice receiving dual therapy [147].

#### 3.1.11. Thioaptamer Targeting Annexin A2

Given the central role angiogenesis plays in cancer progression, therapies have sought to target immature tumor blood vessels, yet have only achieved limited efficacy due to treatment induced-hypoxia. Mangala et al. developed a novel approach for the identification of target thioaptanmers using patient-derived endothelial cells [169]. Thioaptamers specific to tumor associated endothelial cells isolated from ovarian cancer patients were isolated following repeated cycles of negative and positive selection. This unbiased selection method allows for a selection of aptamers that specifically bind to tumor vessels but not to normal vessels. Mass spectrometry data identified a molecular target, the tumor endothelial cell specific membrane protein annexin A2. Treatment with the aptamer/microRNA-inhibitor complex restored tight junction function and improved chemotherapy delivery in orthotopic ovarian cancer mouse models, reducing tumor growth.

#### 3.1.12. Bispecific Aptamers

Bispecific aptamers have been generated to specifically and simultaneously interact with two independent targets. RNA-based bispecific CD44-EpCAM aptamer is capable of blocking CD44 and EpCAM simultaneously by fusing single CD44 and EpCAM aptamers with a double stranded RNA adaptor. Bispecific CD44-EpCAM aptamer suppressed intraperitoneal tumor outgrowth more significantly than individual CD44 and EpCAM aptamers did alone or in combination through enhanced targeting of cancer cells. Bispecific aptamers are well adapted for anti-cancer immunotherapy with minimal toxicity. Co-stimulatory ligand binding of tumor antigen-specific lymphocytes is one immunotherapy approach to boost the antitumor immune response [170]. However, the use of agonistic antibodies such as anti-4-1BB or anti-CD28 have proven difficult due to activation of the lymphocytes expressing the co-stimulatory receptor. In fact, multi-organ toxicity following the administration of agonistic CD28 antibodies such as TGN1412 led to early termination of clinical trial [171]. Liver toxicity is a concern with 4-1BB-mediated treatment of cancer [172]. To alleviate possible adverse effects, bispecific aptamers were developed to bridge between cancer cells and co-stimulatory receptors. For example, bispecific aptamer PSMA-41BB is composed of PSMA and the agonistic 4-1BB aptamers. PSMA binding to cancer cells and 4-1BB binding to the co-stimulatory receptor trigger its activation. Similarly, bispecific aptamers such as VEGF-4-1BB, CD28-MRP1, and CD16a-C-Met have been generated [136,137,138]. These bispecific aptamers have shown higher anti-tumor efficacy relative to non-targeted, co-stimulation reagents or their corresponding monoclonal antibody, achieving the same effect with lower toxicity [136,173,174].

### 3.2. Antidote Aptamer for Controlled Therapy

Eradication of malignant cells is currently the main goal of cancer therapies. Therapeutic efficacy and toxicity are often two sides of the same coin and the balance is fundamental to determining therapeutic efficacy. Humanized monoclonal antibodies have shown circulation half-lives as long as several weeks, enabling less frequent drug administration. Such patient-friendly regimens not only improve quality of life, but are also expected to have enhanced anti-tumor efficacy due to continuous drug exposure. However, unanticipated toxicity from such long-circulating drugs may potentially be prolonged unless otherwise inactivated. Antidote aptamers are a strategy to control the action time of therapeutic aptamers using the corresponding complementary sequence. Hybridization with the antidote leads to a conformational change and loss of target binding. Attempts to control drug action time using this method have been confirmed in animal experiments [175,176]. Rusconi et al. showed that coagulation factor IXa antagonistic aptamer Peg-9.3t’s potent anticoagulant activity was efficiently reversed by administration of the complementary oligonucleotide (antidote oligonucleotide 5–2) within 10 min in human plasma. An antidote oligonucleotide for R9D-14T, a RNA aptamer that binds prothrombin and thrombin pro/exosite I, has been shown to swiftly (<2 min) reverse F9D-14T’s anti-coagulation activity in an assay containing human plasma [176].

## 4. Clinical Trials of Aptamer Application in Oncology

As previously discussed, renal filtration, nuclease degradation, and safety profile present some of the major hurdles for aptamer therapeutics. However, the degree of such challenges may vary with route of administration as well as the targeted site. The best example of an aptamer therapeutic that has overcome these difficulties is pegaptanib, the first only aptamer to receive FDA marketing approval. In 2004, following almost a decade of development and testing, the efficacy of the RNA aptamer commercially known as Macugen^®^ (Eyetech Pharmaceuticals, New York, NY, USA) in treating neovascular age-related macular (AMD) degeneration was confirmed by clinical trial [177]. Through intravitreous administration every 6 weeks over a 48-week course, pegaptanib acts as an antagonist to vascular endothelial growth factor (VEGF), which plays a role in pathologic angiogenesis. In addition to abnormal neovascularization, VEGF contributes to increased vascular permeability and other effects important not only in the development of macular degeneration, but also cancer [9,178,179]. Given the significant role VEGF plays in tumors it was initially thought that pegaptanib may display anti-cancer properties as well. Unfortunately, preclinical models failed to substantiate this hypothesis and the drug has not been tested in a clinical trial for oncology application [29]. The bioavailability of intravitrious injections may contribute to the difference in pegaptanib’s efficacy in AMD and solid tumors, since the former route does not necessitate extended circulation time to reach the target as it does in tumors. Further preclinical studies, however, are underway to address novel ways of improving the biostability of aptamers like pegaptanib [52].

To date, two therapeutic aptamers have made successful transition to clinical trials for oncology, although several ongoing trials have incorporated the development of targeted aptamers for either detection or monitoring into their study design. The first aptamer in clinical trials for cancer treatment was AS1411, a nucleolin-targeting DNA aptamer [100]. Its unique G-rich quadruplex structure as well as pegylation aid the pharmacokinetic profile of AS1411, providing nuclease evasion and an extended half-life [100]. Two clinical trials of AS1411 have assessed safety and efficacy in advanced solid tumors and renal cell carcinoma, respectively (NCT00881244 [180] and NCT00740441 [181]), while a third examined AS1411 in treatment of acute myeloid leukemia (AML) (NCT00512083 [182]). In its initial dose-escalation Phase I clinical study, AS1411 was administered as a continuous intravenous infusion at doses ranging from 1–40 mg/kg/day for 4 to 7 days in up to two cycles of treatment for patients with advanced solid tumors and was found to be well tolerated without serious side effects. Given the promising results of initial clinical assessment, a Phase II trial was conducted in patients with metastatic refractory renal cell carcinoma (RCC), but found the drug to have limited activity in unselected patients, indicating the need for biomarkers for AS1411 responsive tumors. Pre-clinical studies demonstrating that AS1411 targets cell-membrane nucleolin in leukemia cells [183] and results in cell death for acute myeloid leukemia (AML) cell lines [184] indicated its efficacy in hematologic malignancies as well. Another Phase II trial examined the effect of combination therapy with AS1411 and high-dose cytarabine in relapsed/refractory AML patients and found improved response rates among patients receiving combination therapy with continuous infusion of either 10 or 40 mg/kg/day dosing of AS1411 when compared to the control cytarabine only group [185]. Several pre-clinical models are also investigating its role as a potential transport mechanism for other anti-cancer drugs (See the under investigation section), possibly expanding its impact from hematologic malignancies to solid as well.

The second therapeutic aptamer to launch clinical trials for cancer is NOX-A12 (Olaptesed Pegol, NOXXON), an antagonist of the chemokine CXCL12 (or SDF1). Thus far, NOX-A12 has only completed clinical trials for hematologic malignancies, although a new two-part trial (NCT03168139 [186]) is currently recruiting patients with colorectal or pancreatic cancer. NOX-A12 is an L-form RNA aptamer (or Spiegelmer) whose inverted stereochemistry provides nuclear resistance, while PEGylation at the 3′-terminus provides further enhancement of its pharmacokinetic parameters. Pre-clinical models showed that NOX-A12 mobilized white blood cells, hematopoietic stem cells, and progenitor cells in the peripheral blood of mice and healthy human volunteers [104] and Phase I trials (NCT00976378 [187] and NCT01194934 [188]) established its safety profile in healthy volunteers. Pre-clinical models demonstrated that co-treatment of multiple myeloma (MM) cells with NOX-A12 inhibited chemotaxis of MM to bone marrow, inhibiting cell adhesion-mediated drug resistance and sensitizing them to chemotherapy [189]. NOX-A12 also decreased BM niche microenvironment receptivity to MM cells, halting an essential step in disease progression in mouse models [190]. Thus, a Phase II trial (NCT01521533 [191]) comparing single dose intravenous injections of NOX-A12 alone and in combination with bortezomib and dexamethasone (VD) in multiple myeloma (MM) patients who had received previous treatment was performed. Results from this trial indicated that NOX-A12 enhanced the efficacy of VD treatment without increasing treatment toxicity [192]. Pre-clinical models in chronic lymphocytic leukemia (CLL) also showed that NOX-A12 effectively prevented chemotaxis of cancer cells towards CXCL12 and sensitized the cells to chemotherapies in bone marrow stem cell (BMSC) co-cultures [106]. Phase II study (NCT01486797 [193]) testing the efficacy of NOX-A12 in combination with bendamustine and rituximab chemotherapy in patients with relapsed CLL also found improved overall response rates as well as increasing rates of remission in patients treated with NOX-A12 [194]. The aptamer is currently in the recruitment stage of a two-part, phase I/II clinical trial of testing the drug in combination with pembrolizumab in metastatic colorectal and pancreatic cancer (NCT03168139 [186]).

## 5. Conclusions and Future Perspectives

In the era of personalized medicine, targeted therapy has become an integral part of cancer treatment in conjunction with conventional chemo- and radiotherapy, and many molecularly targeted drugs are under investigation and in the drug development pipeline [195]. Aptamers have evolved rapidly in the past 10 years as evidenced by the steep increase in related peer-review publications and US patents, and have been highlighted as the next generation of anti-cancer drug owing to their unique characteristics (high affinity binding, low immunogenicity, ease of synthesis, and chemical modification). A range of molecules involved in tumor progression and metastasis, present at different sites (i.e., circulation, cancer cell, tumor stroma, tumor associated vessel, and pre-metastic vascular niche), have been targeted by aptamers. Pre-clinical investigations of aptamers have shown promising efficacies as well as safety profiles. Creative chemistry has effectively addressed the primary challenges of aptamer therapeutics. Mounting evidence supports the competencies of aptamers for clinical utility in treating cancer, and currently, two anti-cancer aptamers have launched clinical trials. As safe, high-affinity therapeutics, aptamers present an exceptional opportunity to make personalized medicine a reality for more patients with cancer.

## Figures and Tables

**Table 1:** Molecularly targeted anti-cancer aptamers with confirmed in vivo anti-cancer efficacy.

Aptamer Name	Targets	References
ARC126 (RNA) AX102 (RNA)	PDGF-B	Akiyama, Kachi et al., 2006 [96], Sennino, Falcon et al., 2007 [97]
SL (2)-B (DNA) RNV66 (DNA)	VEGF-165	Kaur, Li et al., 2013 [98], Gantenbein, Sarikaya et al., 2015 [99]
AS1411 (DNA)	Nucleolin	Ireson and Kelland 2006 [100], Bates, Laber et al., 2009 [101], Mongelard and Bouvet 2010 [102]
FCL-II (DNA, modified form AS1411)		Fan, Sun et al., 2017 [103]
NOX-A12 (RNA)	CXCL12	Vater, Sahlmann et al., 2013 [104], Liu, Alomran et al., 2014 [105], Hoellenriegel, Zboralski et al., 2014 [106], Zboralski, Hoehlig et al., 2017 [107]
E0727 (RNA) CL428 (RNA) KDI130 (RNA) TuTu2231 (RNA)	EGFR	Li, Nguyen et al., 2011 [8], Esposito, Passaro et al., 2011 [7], Wan, Tamuly et al., 2013 [108], Buerger, Nagel-Wolfrum et al., 2003 [109], Wang, Song et al., 2014 [110]
Trimeric apt (DNA)	HER2	Kim and Jeong 2011 [111], Mahlknecht, Maron et al., 2013 [112]
PNDA-3 (DNA)	Periostin	Lee, Kim et al., 2013 [113]
TTA140,41 (DNA) GBI-1042 (DNA)	TN-C	Hicke, Stephens et al., 2006 [114], Daniels, Chen et al., 2003 [115], Hicke, Marion et al., 2001 [116]
NAS-24 (DNA)	Vimentin	Zamay, Kolovskaya et al., 2014 [117]
YJ-1 (RNA)	CEA	Lee, Han et al., 2012 [118]
AGE-apt (DNA)	AGE	Ojima, Matsui et al., 2014 [119]
A-P50 (RNA)	NF-κB	Mi, Zhang et al., 2008 [120]
GL21.T (RNA)	Axl	Cerchia, Esposito et al., 2012 [121]
OPN-R3 (RNA)	OPN	Mi, Guo et al., 2009 [122], Talbot, Mi et al., 2011 [123]
AGC03 (DNA) cy-apt (DNA)	HGC-27	Zhang, Zhang et al., 2014 [124], Cao, Yuan et al., 2014 [125]
BC15 (DNA)	hnRNP A1	Li, Wang et al., 2012 [126]
A9g (RNA)	PSMA	Dassie, Hernandez et al., 2014 [127]
ESTA (DNA)	E-selectin	Mann, Somasunderam et al., 2010 [128], Kang, Hasan et al., 2015 [129], Kang, Blache et al., 2016 [130], Morita, Kamal et al., 2016 [62]
M12-23 (RNA)	4-1 BB	McNamara, Kolonias et al., 2008 [131]
OX40-apt (RNA)	OX40	Dollins, Nair et al., 2008 [132], Pratico, Sullenger et al., 2013 [133]
CD28-apt (RNA)	CD28	Pastor, Soldevilla et al., 2013 [134]
Del60 (RNA)	CTLA-4	Santulli-Marotto, Nair et al., 2003 [135]
PSMA-4-1BB-apt (RNA)	PSMA/4-1BB	Pastor, Kolonias et al., 2011 [136]
CD16*α*/c-Met-apt (RNA)	CD16*α*/c-Met	Eder, Vande Woude et al., 2009 [137]
VEGF-4-1BB apt (DNA)	VEGF/4-1BB	Schrand, Berezhnoy et al., 2014 [138]
MP7 (DNA)	PD-1	Prodeus, Abdul-Wahid et al., 2015 [139]
aptPD-L1 (DNA)	PD-L1	Lai, Huang et al., 2016 [140]
R5A1 (RNA)	IL10R	Berezhnoy, Stewart et al., 2012 [141]
CL-42 (RNA)	IL4Rα	Roth, De La Fuente et al., 2012 [142]
CD44-EpCAM aptamer (RNA)	CD44/EpCAM	Zheng, Zhao et al., 2017 [143]
TIM3Apt (RNA)	TIM3	Hervas-Stubbs, Soldevilla et al., 2016 [144]
CD40apt (RNA)	CD40	Soldevilla, Villanueva et al., 2015 [145]
AptCTLA-4 (DNA)	CTLA-4	Huang, Lai et al., 2017 [146]
AON-D21 l-Aptamer (RNA/DNA)	C5a	Ajona, Ortiz-Espinosa et al., 2017 [147]
